# Learning About Credition: Exploring the Barriers Between Basic and Applied Research

**DOI:** 10.3389/fpsyg.2022.943376

**Published:** 2022-06-29

**Authors:** Davor Madzarevic

**Affiliations:** Institute of Catechetics and Religious Education, University of Graz, Graz, Austria

**Keywords:** learning about credition, learning barriers, language use, priors, instruction about credition

## Introduction

This paper offers the first insights into a research project in progress: “Blind Spot Credition: Bridging the gap between basic research and application” at the Karl-Franzens University of Graz. The intended project is part of the Credition Research Project (Angel, 2022) and has a special focus on religious education, but we understand our research as paradigmatic and applicable for any kind of education dealing with the topic of belief and believing in public schools. The project has in mind the situation of teachers in public schools and has the intention to deal with learning barriers in the first approach to credition. An important aim of the project is to detect, define and analyze barriers that prevent “newcomers” (in this case teachers or school children, who haven't heard or learned about creditions so far) when encountering creditions for the first time—be it as an idea, as a concept, as a model or in theoretical debates. We argue that addressing this issue helps to bridge the gap between basic and applied research of the credition research project.

For the empirical part, we have chosen teachers of religious education in public schools as our target group. We intend to start a survey that is international (schools from Croatia, Germany, and Austria are involved in the cooperation network) and will nevertheless provide comparable results. A broader empirical database is not yet available, but we have data from pre-test studies that have not yet been published.

In this paper, I will present the theoretical background and first results which have been influencing the actual research perspectives.

## Learning and Learning-Theories

Learning and learning theories are fundamental when it comes to any matter that needs to be mastered hermeneutically. We do not intend to contribute to the theory-building of learning theories. When we talk about learning, we mean a strategy on how to overcome super complex material burdened with various hermeneutic barriers. Our basic assumption is that without detecting and deciphering those barriers our learning approach to credition will be finished before it effectively starts. We are interested in the emotional foundations of barriers and their learning implications.

We also want to understand which ways or means are the most appropriate to help school children and their teachers to understand the basic concept of credition. We want to figure out how pathways can be developed to make credition attractive to them, even though they do not and cannot know what to expect.

Finally, in an attempt to identify the barriers, we are interested in the research of timeline: Starting condition, learning steps, their challenges, and the end condition.

## Different Starting Conditions

Starting condition refers to the three different levels from which our research proceeds: school children, teachers, and science.

Based on pre-test experiences, school children most often find the material from the credition research project uncomfortable because they automatically associate it with religion, but also with prejudices related to, e.g., philosophy. They often complain about the problem of hermeneutics and the complexity of the credition topic as well. Teachers also encounter a variety of issues in learning about credition. They mostly find a tense relationship between motivation, energy investment in learning, and the benefits of credition. Learning about credition does not progress smoothly in the scholarly fields either. Those who come from the Humanities often find it daunting that there is a wide range of interdisciplinary knowledge about credition. Different disciplines are included: philosophy, cognitive science, and natural science. And sometimes it can be difficult for people to understand that there is conceptual knowledge as the basis for understanding the model.

There are also a lot of incompatibilities and cross relations between different starting positions. For example, when speaking about beliefs, school children or teachers may have in mind religion but not epistemology. Epistemologists may be thinking about philosophical issues, i.e., justification but not the need to make these debates accessible, etc.

One of the first empirical pre-test results is that there are some difficulties and complications in the understanding of credition, and therefore finding a starting point for the research represents a crucial challenge. We assume that the starting point cannot be credition itself, but human consciousness. Exploring associations of “belief” from pre-test studies seemed fruitful to our research reflections. In the pre-tests, we found the word association method (Kent and Rosanoff, 1910) helpful. In our research approach, we will highlight parameters that indicate credition as a blind spot for learning. One of these is how language is used when talking about beliefs.

## Language Use

Pre-test studies show that language use contributes to barriers in first learning about credition. Thus, in a pre-test study conducted in a primary school in Bosnia and Herzegovina, with the sample of 27 pupils, mostly the following associations to the term “belief” popped up: God (came up even 15 times), Jesus, love, hope, holiness, prayer, etc. When asked what “belief” means to them, the students mostly answered: “Belief means to have belief in someone, for example, to trust your parents or to believe in God” or “It means a lifestyle in which we are devoted to God”. In two other similar questionnaires more than half of the responses indicated linking believing with religion. These associations and definitions of belief have certain implications for at least the three main characteristics of understanding the belief:

1. Belief as a noun.
2. Belief as religious belief.
3. Belief concerning (religious) content.

This also suggests that learning about credition has to do with the students' different backgrounds in learning subjects, their predispositions to learn, their worldviews, and especially their attitudes toward “beliefs”. Specifically, this means that how the language is used indicates cognitive assumptions of prior knowledge and information that can influence the adoption of new information, knowledge, and new conclusions, which in psychology are referred to as “priors” (Tobias, 1994; Dochy, 1996).

## Priors

In our research we have already identified some consequences of different priors related to the above-mentioned common use of beliefs:

(1) In a broader philosophical sense, the question of belief is embedded in a long tradition of Western thinking and has produced a rich and overwhelmingly broad literature base over about 2,500 years. Therefore, one may get the impression that belief is a well-defined phenomenon, but newer interdisciplinary approaches to the processes of believing deny such an understanding and show that belief is an ill-defined phenomenon.
(2) Another prior may be identified in an approach to belief which seems to be especially influential in neuropsychiatry and psychology. Belief seems to be associated with pathology. Thus, belief can be related to neurosis or delusion (McCauley and Graham, 2020). This can cause a variety of problems because linking pathology to belief can automatically cause a negative attitude toward any approach to credition.
(3) The everyday use of language, also demonstrated in pre-test studies, shows a close connection between understanding “belief” as a religious belief. Evidence for this can be also found on the theoretical level in the credition literature: “No other concept relevant to understanding human behavior is as deeply tied to religion as belief” (Angel et al., 2017, p. 5). This is of course highly problematic for a correct understanding of credition that is not limited just to religion.
(4) Another prior is the very frequent use of “belief” both in everyday speech and in scientific discourse as a noun. For instance, the predominant use of nouns like “formation of belief” (Langdon and Connaughton, 2013) or “dynamics of belief” (Forrest, 1986) work against conceptualizing believing processes as having a fluid character (cf. Angel, 2017, p. 19).
(5) In everyday understanding, beliefs are often content-oriented. Testimonial beliefs (I believe in) and fiduciary beliefs (to have faith in) are often expressed here. Such a prior significantly reduces the likelihood of a proper understanding of the process of believing.

## Discussion

### Barriers to an Approach to Credition

The core task of the project in progress, which this paper intends to present, is to illuminate barriers that prevent a correct understanding of the fluidity of believing in the school context. To achieve the desired goal, we use already existing scientific parameters such as:

epistemology, which focuses predominantly on the question of justification (Runehov, 2017); philosophy of mind, which focuses predominantly on the nature of beliefs (Visala and Angel, 2017) and eliminativism, which claims that belief should not be a matter of scientific debate (Stich, 1996), etc.

These parameters influence the several theoretical levels that coincided with some results which were obtained from pre-test studies:

(1) Process of believing is a blind spot in the mind; therefore, no initial associations are pointing to the fluidity of beliefs.
(2) Belief is initially marked as a noun in the mind. Therefore, the paradigmatic shift from belief to credition seems to be irrelevant.
(3) Credition has something to do with belief. Belief is immediately associated with religion, therefore credition is monopolized by religion. The same confusion is with the term “religiosity” (cf. Angel in this volume) which seems instinctively associated with religion. This is counterproductive for any understanding of the processes of believing. From a cognitive neuroscience point of view, it must be stated that creditions do not take place in religions but rather in humans when they develop and live their religiosity.
(4) Belief has something to do with knowledge, but the relevance of epistemological discussions seems to play a significant role for the newcomers.
(5) The initial notion of cognitive science and neuroscience is the neglect of the mind. Even if belief is understood as an inner process, then biological and cognitive science background knowledge is required and already provided by science.

### Barriers to the Instruction About Credition

Addressing barriers in this project has several implications for a particular strategy for instruction about credition. In our approach, we trace the following strategies. First, it is necessary to draw attention to credition as a blind spot. Second, it is necessary to make the blind spot attractive enough to provoke energy and exertion for learning. Finally, it is important to make attractive the benefit of learning about credition for pupils. A special emphasis could be placed on those aspects where the topic of belief comes directly or indirectly into play, such as the role of creditions in dealing with catastrophes (Sugiura, 2017) or the connection between creditions and identity development (Colagè and Gobbi, 2017) or the influence of creditions on decision-making (Hick et al., 2020), etc.

Following these three important pre-steps, it is possible to anticipate further strategic steps for learning. Explaining the scientific background of the credition concept (cf. Angel in this volume) can represent one of the initial steps. In developing different strategic steps, setting clear goals for learning about credition should not be neglected as well. Determining the amount of information and knowledge within a certain time frame to achieve the desired goals is therefore of primary interest in the learning strategy. At the same time, the balance between investing energy and achieving set goals should be kept in mind.

Identifying and analyzing barriers in the approach to credition enables didactic creativity in presenting the concept and model to pupils as well. In doing so, teachers should pay attention to avoiding already established barriers and provide students with the most unobtrusive approach to credition. They could also use various didactic methods and means of digital learning and teaching when presenting the concept and the model. This would make the matter they are learning as interesting as possible. For that aim, E-Learning Methodologies and Tools (Wang, 2012) based on Cognitive Load Theory (Sweller et al., 2019) could be useful here. Finally, teachers should motivate and enable students to work individually with the model of credition and encourage communication of their personal experiences and reflections in working with the model (cf. Mitropoulou et al., 2018).

In the end, developing a learning and teaching strategy should help to integrate credition more successfully into the future school context. Some of the theoretical steps presented here are part of this paper, but we can only evaluate the results of the study after they are available.

## Author Contributions

The author confirms being the sole contributor of this work and has approved it for publication.

## Funding

This article was funded by Dr. Rüdiger Seitz, via the Volkswagen Foundation, Siemens Healthineers, and the Betz Foundation.

## Conflict of Interest

The author declares that the research was conducted in the absence of any commercial or financial relationships that could be construed as a potential conflict of interest.

## Publisher's Note

All claims expressed in this article are solely those of the authors and do not necessarily represent those of their affiliated organizations, or those of the publisher, the editors and the reviewers. Any product that may be evaluated in this article, or claim that may be made by its manufacturer, is not guaranteed or endorsed by the publisher.
